# Selective cytotoxicity of vanadium complexes on human pancreatic ductal adenocarcinoma cell line by inducing necroptosis, apoptosis and mitotic catastrophe process

**DOI:** 10.18632/oncotarget.19454

**Published:** 2017-07-22

**Authors:** Szymon Kowalski, Stanisław Hać, Dariusz Wyrzykowski, Agata Zauszkiewicz-Pawlak, Iwona Inkielewicz-Stępniak

**Affiliations:** ^1^ Department of Medical Chemistry, Medical University of Gdansk, Gdansk, Poland; ^2^ Department of General, Endocrine and Transplantation Surgery, Medical University of Gdansk, Gdansk, Poland; ^3^ Faculty of Chemistry, University of Gdansk, Gdansk, Poland; ^4^ Department of Histology, Medical University of Gdansk, Gdansk, Poland

**Keywords:** vanadium complexes, pancreatic cancer, necroptosis, mitotic catastrophe, autophagy

## Abstract

The pancreatic cancer is the fourth leading cause of cancer-related death and characterized by one of the lowest five-year survival rate. The current therapeutic options are demonstrating minimal effectiveness, therefore studies on new potential anticancer compounds, with non-significant side effects are highly desirable. Recently, it was demonstrated that vanadium compounds, in particular organic derivatives, exhibit anticancer properties against different type of tumor as well as favorable biodistribution from a pancreatic cancer treatment perspective.

In this research, we showed selective cytotoxic effect of vanadium complexes, containing phenanthroline and quinoline as an organic ligands, against human pancreatic ductal adenocarcinoma cell line (PANC-1), compared to non-tumor human immortalized pancreas duct epithelial cells (hTERT-HPNE). Results exhibited that vanadium complexes inhibited autophagy process in selective cytotoxic concentration as well as caused the cell cycle arrest in G2/M phase associated with mitotic catastrophe and increased level of reactive oxygen species (ROS). Moreover, in higher concentration, vanadium derivatives induced a mix type of cell death in PANC-1 cells, including apoptotic and necroptotic process.

Our investigation emphasizes the anticancer potential of vanadium complexes by indicating their selective cytotoxic activity, through different process posed by alternative type of cell deaths to apoptosis-resistant cancer cells. Further studies supporting the therapeutic potential of vanadium in pancreatic cancer treatment is highly recommended.

## INTRODUCTION

The pancreatic cancer is the fourth leading cause of cancer-related death. In spite of the fact that pancreatic cancer is much less common than the breast or bowel tumor, it is characterized by one of the lowest 5-year survival rate. Roughly 3% of patients survive over five years, when the usual is under six months [1–4]. More than 80% of patients are identified with locally advanced and metastatic stage, unable to surgical resection. This is caused by delayed diagnosis and lack of specific urine or blood markers which could be used to identify patients with increased risk [5–7]. The localization of tumor also has diagnostic and clinical significance. The tumor located in the head region of pancreas because of biliary impediment are identified relatively early. In contrast, the tumors spotted in the body and tail of pancreas stay asymptomatic until late stage of disease [7]. Unfortunately, even for the 20% patients with a resectable tumor, prospect of long-term survival is still unavailable. The incidence of pancreatic cancer is highly diverse across regions and populations, suggesting the influence of genetic and environmental factors. Furthermore, pancreatic tumors is strongly age-dependent. The substantial population of patients are over the age of 60. The other danger components include obesity, smoking, alcohol and high utilization of processed meat [8, 9]. About 90% of pancreatic tumor is derived from the ductal epithelium of pancreas and termed as pancreatic ductal adenocarcinoma (PDAC) [1].

Although the current chemotherapy regimens improved survival results, therapeutic options are demonstrating minimal effective. The recent clinical trials have shown only slight improvements in total survival [10]. In case of advanced PDAC, gemcitabine has been better to 5-fluorouracil in overall survival (5.65 compared to 4.41 months) and has shown substantial clinical benefit in relieving of disease symptoms [11]. In 2011 was demonstrated new therapeutic strategy including four components: oxaliplatin, irinotecan, 5-fluorouracil and folinic acid, consequently termed as FOLFIRINOX. The clinical investigation demonstrated that FOLFIRINOX has much better survival rate than gemcitabine (11.1 compared to 6.8 months), but higher survival is correlated with significant toxicity and decreased quality of life [12].

It is well known that cancer cells are able to avoid the cell death process and it is considered to one of the hallmarks of cancer [13]. The clinical implications of the evasion of cell death cause the development of cell resistance during chemotherapeutic treatment. Therefore, growing interest in mechanisms of different cell deaths, such as apoptosis, autophagy, necroptosis, endoplasmic reticulum stress and others has allowed to discover new regulating process, interfering with death pathways and which can constitute a promising molecular target for cancer cells [14–19].

Other hallmarks of cancer cells including genome instability and mutation [13]. Defective mitosis leads to abnormal number of chromosomes: aneuploidy or tetraploid cells. It is a common feature of cancer cells [20]. Furthermore, many cancer cells are deficient in the G1 checkpoint, causing fail to arrest cell cycle in the G1 phase and a temporary accumulation cells in the G2 cycle. However, G2 checkpoint is also partially damaged in tumor cells, as a consequence, it leads to die upon entrance into mitosis [21]. This process is called a mitotic catastrophe or mitotic death and could constitute a promising target for antitumor drugs, due to more intense and longer-lasting effect *in vivo* [22].

Necroptosis is one of the programmed necrosis form induced by ligand death receptor such as TNFα, FasL and TRAIL. As well as necrosis, necroptosis process is characterized by morphological changes including loss of plasma-membrane integrity, cell and organelle swelling and ultimately cell lysis [23, 24]. As some research suggests, necroptosis can be induced as an alternative cell death for apoptotic pathway in the case of pharmacological inhibition or genetic ablation of apoptosis process, making it promising target in apoptotic resistance cells [25, 26].

Autophagy is term as type II programmed cell death. Division of autophagy process based on varying mechanism of delivery loads to lysosomes and the most characteristic morphological feature of autophagy is formation of the autophagosome, double-membrane autophagic vacuoles containing mitochondria, endoplasmic reticulum, ribosomes and protein designed to degradation [27, 28]. From a pancreatic cancer perspective, autophagy plays a complex role in the development of tumor. Numerous studies have shown both tumor-suppressive and pro-tumorigenic roles [29–31]. On the other hand, higher basal levels of autophagy in PDAC cells make their easier survival under stressful condition like hypoxia, nutrient deprivation or chemotherapy [32].

Vanadium compounds, in particular organic derivatives, exhibit a numerous biological activities, including anticancer properties [33]. The molecular mechanisms responsible for their anticancer effect including generation of ROS, DNA damage, as well as alteration of the spindle proteins like tubulin or actin and cellular organelles such as mitochondria or lysosomes [33, 34]. In rats treated vanadium salt or organic derivatives, vanadium was detected in bone, kidney, spleen and also in pancreas [35–37]. Through their favorable biodistribution and complex mechanism of anticancer activity, vanadium compounds seem to be very attractive molecules, that would be used for the treatment of pancreatic cancer.

Therefore, the aim of our investigation was to perform a preliminary screening of seven synthesized vanadium complex, for their cytotoxic activity against human pancreatic ductal adenocarcinoma cell line, compared to non-tumor immortalized pancreas duct epithelial cells. Subsequently, for chosen vanadium compounds with a selective activity, we assessed their molecular mechanisms with particular focus on type of cell death, including: apoptosis, necroptosis and autophagy.

## RESULTS

**Figure 1:**
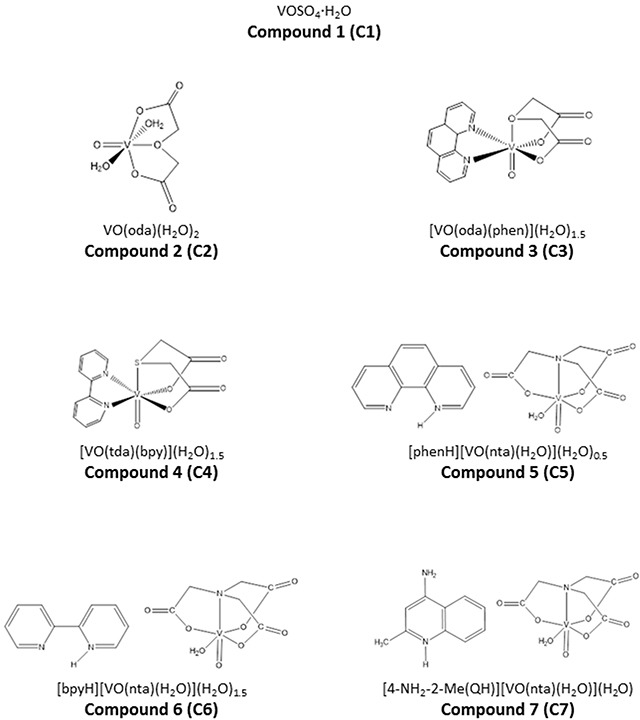
The chemical structures of synthesized vanadium complexes

In the present study we have selected seven vanadium complexes (C1-C7), the structure shown in Figure 1, to evaluate their cytotoxicity against human pancreas ductal adenocarcinoma cells *in vitro*.

C1 is a pure vanadium salt (VOSO_4_), whereas C2 consists of oxydiacetate complex. C3 and C4 contain covalently bound organic ligands: phenanthroline and bipyridine, respectively. In case of C5-C7 compounds, organic ligands (phenanthroline, bipyridine and 4-amino-2-methylquinoline) are connected as counterions.

### Cytotoxic activity of vanadium complexes

The MTT assay was used to evaluate the cytotoxicity effect of seven vanadium complexes (C1-C7) on human pancreas ductal adenocarcinoma cell lines (PANC-1) after 48 h incubation in the range of 1 to 100 μM. In order to determine selective activity against cancer cells, it was used immortalized pancreas duct cells (hTERT-HPNE).

**Figure 2:**
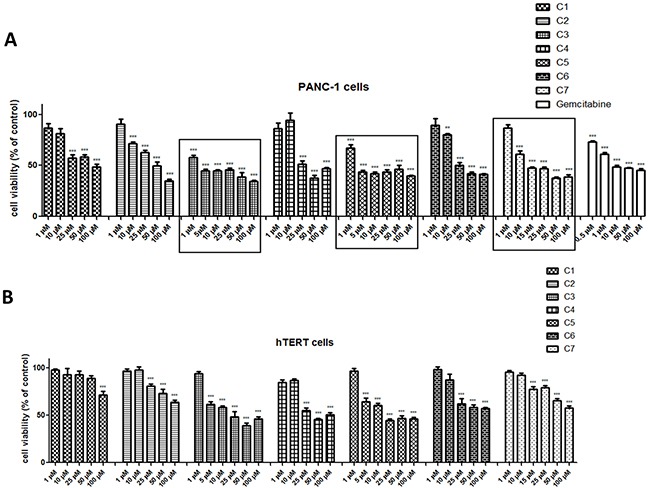
Viability of pancreas ductal adenocarcinoma cell lines (PANC-1) **(A)** and immortalized pancreas duct cells (hTERT-HPNE) **(B)** after treatment with vanadium complexes and gemcitabine. The cells were exposed to seven vanadium complexes (C1-C7) and gemcitabine in the range of 0.5 to 100 μM for 48 h. Data are mean ± SD of 3 separate determinations. *p<0.05; **p<0.01; ***p<0.001 as compared with untreated cells.

Figure 2A and 2B illustrated that tested compounds exhibited cytotoxic effect in a concentration-dependent manner. The Vanadium(IV) sulfate (C1), without any organic components, showed selective cytotoxic effect against PANC-1 at 25 μM and 50 μM concentration, which significantly decreased cancer cell viability (p<0.001).

For three compounds, exhibiting the highest and selective cytotoxicity (Figure 2 and Table 1), dermination of cell viability was repeated for a different range of concentration on PANC-1 and hTERT-HPNE cells (Figure 2).

**Table 1:** Cytotoxic activity of vanadium complexes and gemcitabine against PANC-1 cells after 48 h of treatment. Data are expressed as mean ±SD of 3 separate determinations

Compound	logIC_50_±SD
Compound 1 (C1)	1.92 ± 0.12
Compound 2 (C2)	1.66 ± 0.04
Compound 3 (C3)	0.52 ± 0.28
Compound 4 (C4)	1.66 ± 0.12
Compound 5 (C5)	1.10 ± 0.11
Compound 6 (C6)	1.61 ± 0.07
Compound 7 (C7)	1.47 ± 0.07
Gemcitabine	0.66 ± 0.36

**Figure 3:**
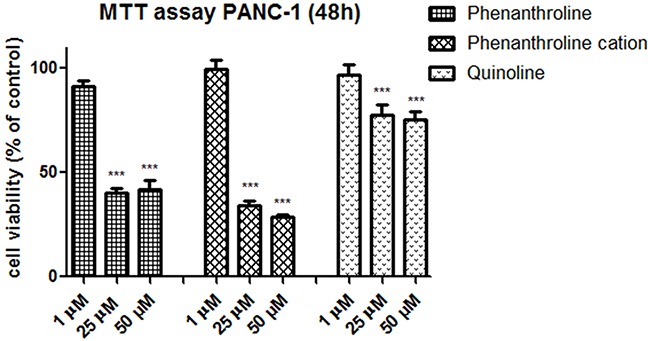
Effect of organic compound: phenanthroline, phenanthroline cation form and quinolone on cell viability, after incubation for 48 h The viability was measured by MTT assay, against pancreas ductal adenocarcinoma cell lines PANC-1. Data are mean ± SD of 3 separate determinations. ***p<0.001 as compared with untreated cells.

The selective impact of C3 and C5 on the cancer cells viability has been observed at concentration - 1 μM, C7 - at concentration of 10 μM (as indicated on Figure 2). However, these complexes of vanadium were effective at all concentrations from 1 to 100 μM and significantly reduced PANC-1 cell viability (p<0.001). Moreover, tested complexes at non-selective concentrations were less cytotoxic on hTERT-HPNE cells after 48 h of exposure. Gemcitabine, commonly used in pancreatic cancer treatment [11], was used as a positive control (Figure 2A) and significantly inhibited cell viability already at concentration of 0.5 μM. The C3 with log IC_50_: 0.52 ± 0.28 exhibited similar cytotoxicity to that of gemcitabine with log IC_50_ of 0.66 ± 0.36 in PANC-1 cells (Table 1). We have also assessed effect of organic components: phenanthroline as cation, quinoline and phenanthroline, which are part of molecular structure of C5, C3 and C7 compound, respectively (Figure 3) on cells viability. Phenanthroline and its cation forms, did not exhibit a significant decline in PANC-1 cells viability at a concentration of 1 μM. Furthermore, the quinolone showed much lower cytotoxic effect against cancer cells compared with C7 at 25 μM and 50 μM concentration.

### Antiproliferative activity of vanadium complex

**Figure 4:**
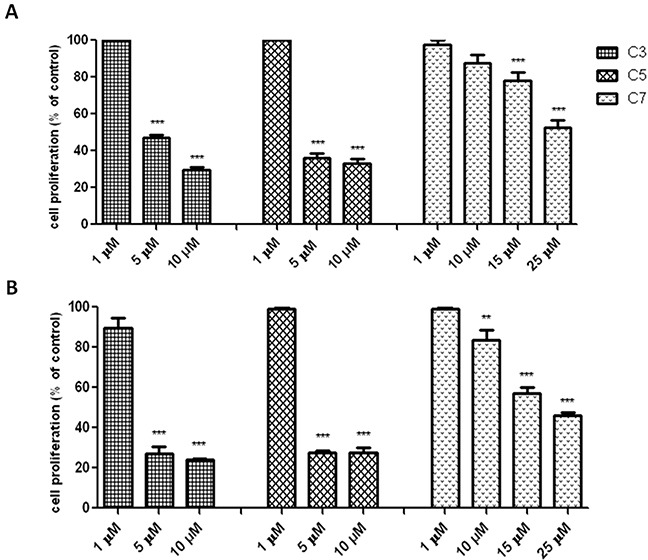
Antiproliferative effect of vanadium compounds against pancreas ductal adenocarcinoma cell lines PANC-1 Inhibition of PANC-1 cell proliferation after 48 **(A)** and 72 **(B)** h of treatment with vanadium compounds detected by quantitative ELISA analysis of BrdU incorporation. Data are mean ± SD of 3 separate determinations. **p<0.01; ***p<0.001 as compared with untreated cells.

The antiproliferative activity of vanadium complex was determined by using the BrdU incorporation assay. It is sensitive test to measure directly the level of DNA synthesis. Quantitative analysis of BrdU assay (Figure 4) indicated that the amount of DNA synthesis was significantly reduced by compounds 3, 5 and 7 in a concentration-dependent manner. In contrast to compound 3 and 5, the compound 7 inhibits significantly DNA synthesis in a time dependent-manner.

### ROS generation

**Figure 5:**
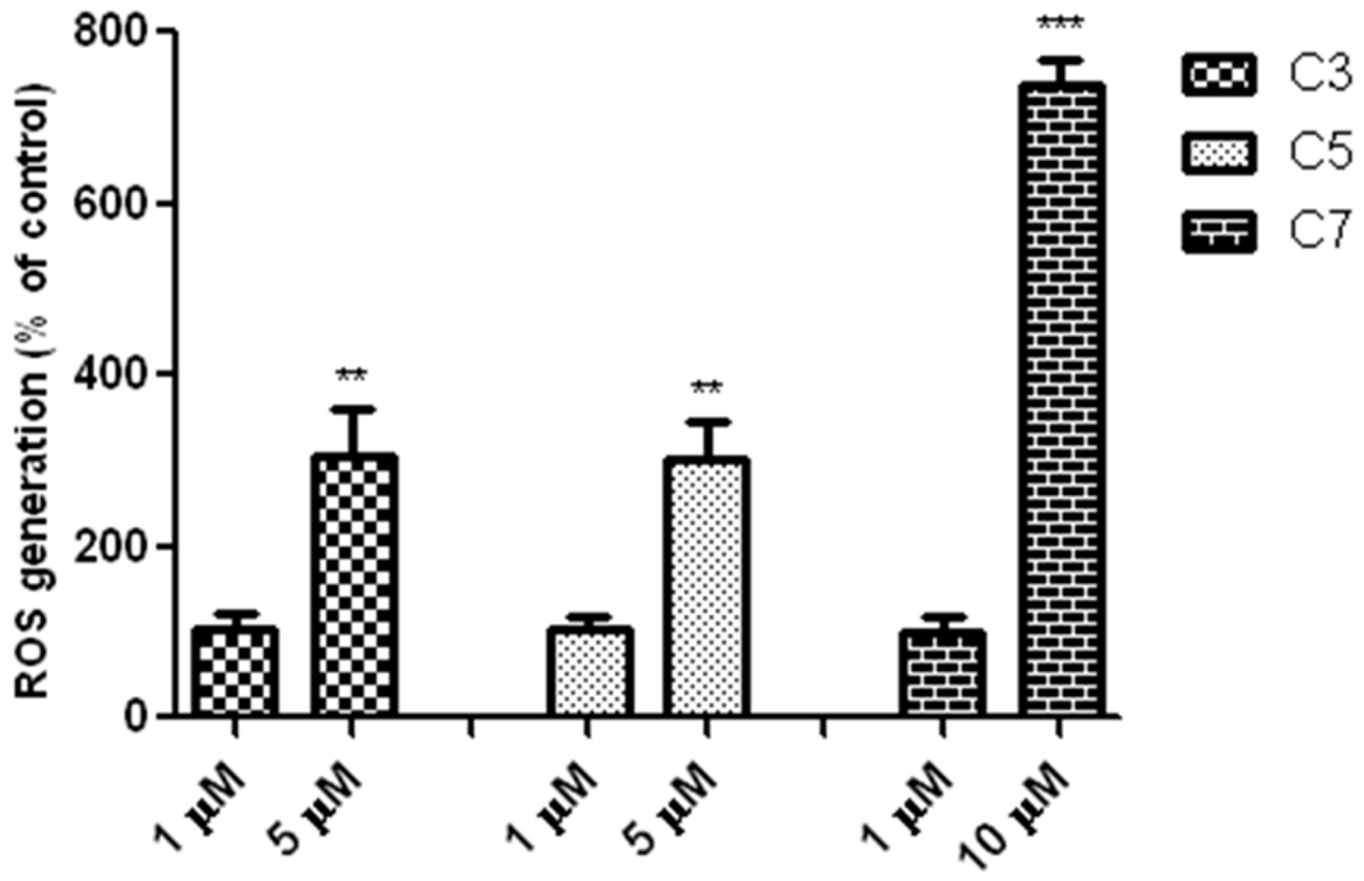
Vanadium complexes increased level of ROS in PANC-1 cells after incubation for 48 h Data are mean ± SD of 3 separate determinations. **p<0.01; ***p<0.001 as compared with untreated cells.

To explore molecular mechanisms underlying the cytotoxicity of C3, C5 and C7 vanadium complexes, it was measured level of ROS (Figure 5).

All investigated compounds induced a significant increase of ROS generation in a time- and concentration-dependent manner. However, only C7 complex at selective concentration of 10 μM increased level of ROS and the increase was about seven times more in comparison to control cells.

### The cell cycle analysis

**Figure 6:**
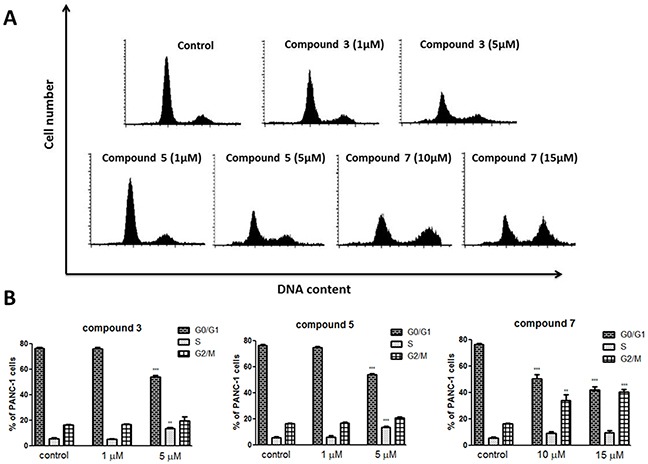
The cell cycle distribution of PANC-1 cells treated with vanadium complexes C3, C5 and C7 after 48 h of incubation **(A)** Representative histograms. **(B)** The percentage of cells in each phase. **(C)** Cell population in sub-G1 fraction. Results are given as mean ± SD of 3 separate determinations. **p<0.01; ***p<0.001 as compared with untreated cells.

Next, we have determined a cell cycle progression in PANC-1 cells by using flow cytometry. The experimental results (Figure 6) indicated that C3 and C5 compounds at concentration of 1 μM did not cause changes in the cell cycle progression compared with control.

However, both compounds at concentration of 5 μM reduced significantly population of cells in the G1 phase, while fraction of sub-G1 cells (representing mainly the apoptotic cells) increased from 0.84% in control cells to 8.65% and 7.05% in cells treated by C3 and C5, respectively (Figure 6). Compound 7 caused accumulation of PANC-1 cells in the G2/M phase, both at 10 μM and 15 μM concentration (34% and 40% compared with 16% in control), when a slight increase of sub-G1 cells population to 4% was observed only at 15 μM concentration (Figure 6).

### Determination of type of cell death

**Figure 7:**
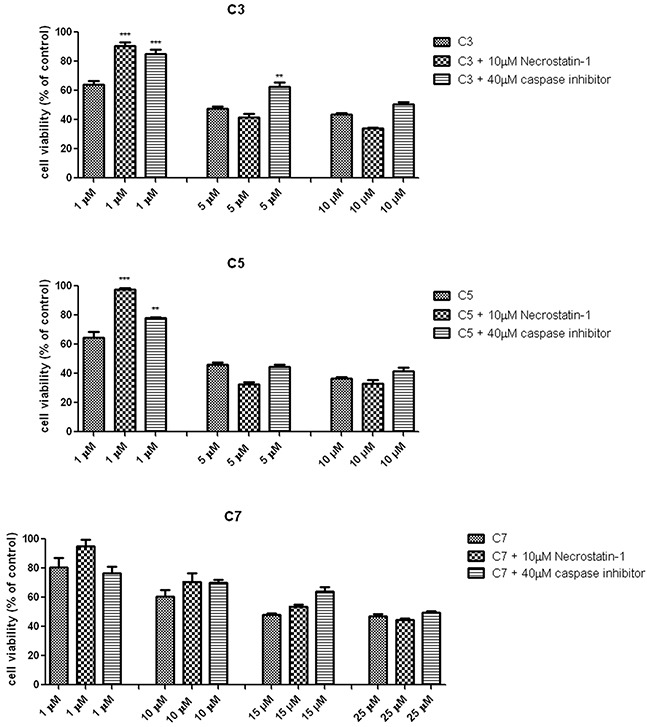
Protective effect of necrostatin-1 and caspase inhibitor on vanadium complexes-induced decrease in PANC-1 cells viability. Cells has been pre-treated with 10 μM Necrostatin-1 and 40 μM caspase inhibitor before exposure to vanadium complexes for 48 h Cell viability was determined by MTT assay. Data are mean ± SD of 3 separate determinations. **p<0.01; ***p<0.001 vanadium complexes-treated cells v/s vanadium complexes-treated cells in the presence of necrostatin-1 or caspase inhibitor.

In order to evaluate whether the decrease of pancreas ductal adenocarcinoma cells viability were induced by apoptotic or necroptosis pathway, the caspase inhibitor (Z-VAD-FMK) and necrostatin-1 had been added to the cells before exposure to vanadium complexes. The Z-VAD-FMK is a general caspase inhibitor, which are necessary to execution of apoptotic process. The necrostatin-1 inhibits RIP1 kinase activity, which is required to trigger necroptosis pathway. As Figure 7 shows, either necrostatin-1 or caspase inhibitor significantly reduced cytotoxic effect of 1 μM C3 and C5 vanadium complexes.

**Figure 8:**
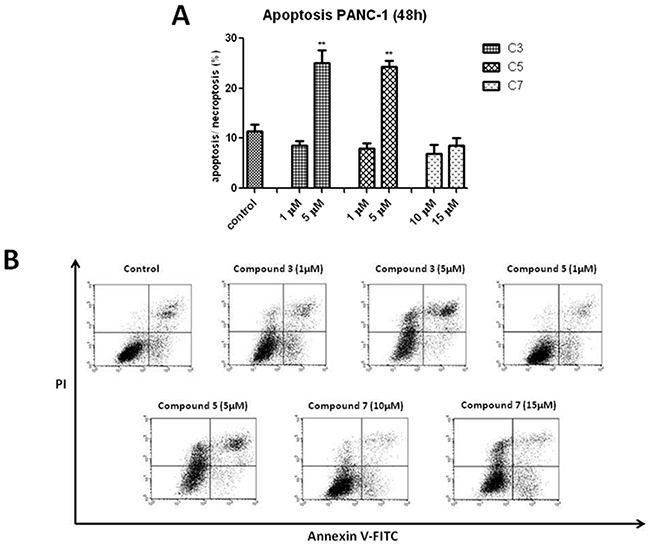
Induction of apoptosis/necroptosis in PANC-1 cells treated with vanadium complexes for 48 h The analysis was performed using flow cytometry. **(A)** Percentage of late apoptotic or necroptotic cells (Annexin V+/PI+). Data are mean ±SD of 3 separate determinations. **p<0.01 as compared with untreated cells. **(B)** Representative bivariate histograms of the Annexin V/PI staining.

In contrast to C3 and C7, neither Necrostatin-1 nor caspase inhibitor had a significant effect on C7-induced cells death. Considering the protective effect of caspase inhibitor, vanadium complexes-treated cells was dual staining with propidium iodide (PI) and Annexin V. This assay is based on the process, when phosphatidylserine relocate from inner cell membrane into the cell surface in the early stage of apoptosis. In contrast, PI can enter into the cells after loss of membrane integrity. Therefore, dual staining with Annexin V/PI allows to determine unimpaired cells (Annexin V−/PI−), early apoptotic cells (Annexin V+/PI−), late apoptotic or necroptotic cells (Annexin V+/PI+) and primary necrotic cells (Annexin V−/PI+), which represents non-programmed type of cell death. We have detected a significant increase in AnnexinV+/PI+ population of PANC-1 cells treated with C3 and C5 at 5 μM concentration (Figure 8). However, a statistically significant increase in (Annexin V+/PI−) population has not been observed.

None of investigated vanadium complexes induced a significant elevation in early apoptosis or primary necrotic population of PANC-1 cells (data not shown).

**Figure 9:**
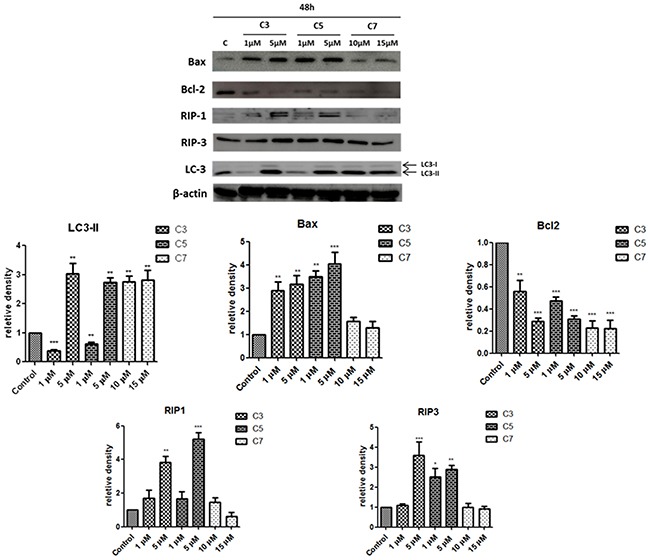
Western blot analysis of protein expression involved in apoptosis, necroptosis and autophagy process in PANC-1 cells exposed to vanadium complexes for 48 h β-actin was used as internal control. Data are mean ±SD of 3 separate determinations. *p<0.05; **p<0.01; ***p<0.001 as compared with untreated cells.

Subsequently, we have evaluated the levels of selected proteins related to different type of cell death. According to Figure 9, the level of proapoptotic protein Bax was increased in cells exposed to C3 and C5 in a concentration-dependent manner, whereas Bcl-2 level was decreased after incubation with C3 and C5 as well as C7. In addition, the level of RIP1 as well as RIP3 kinases increased after treatment with 5 μM of C3 and C5 and slightly - with 1 μM. Interestingly, LC-3 protein, which is a marker of autophagy process, decreased in PANC-1 cells treated with selective (1 μM) concentration of C3 and C5 compounds while after treatment with higher concentration - 5 μM the level of LC3-II increased when compared to control cells. The C7 compound at all investigated concentrations caused an increase of LC3-II protein level. LC3-I is a cytosolic form of LC protein whereas LC3-II is conjugated to phosphatidylethanolamine of the autophagosomal membrane. The amount of LC3-II is correlated with the number of autophagosomes present in cell [38].

**Figure 10:**
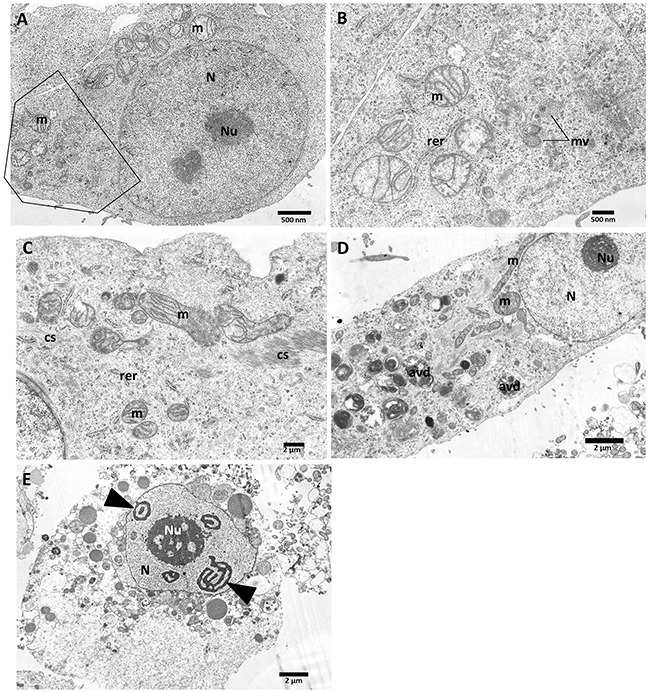
Ultrastructural features of PANC-1 cells incubated with vanadium complexes: C3 and C5 for 48 h Control **(A, B)**; C3- 1μM **(C)**; C3- 5μM **(D)**; C5- 5μM **(E)**. (cs) - microfilaments; (m) - mitochondria;(N) - nucleus; (Nu) - nucleolus; (avd) - late autophagic vacuoles. The scale bar (2μm or 500 nm) is indicated at the bottom right of each image.

Also, the transmission electron microscopy (TEM) has been performed in order to investigate the influence of vanadium compounds on the PANC-1 cells morphology and their organelles. Untreated PANC-1 cells (Figure 10A, B-magnified boxed area) show the typical morphology of adherent cells in TEM: with euchromatic nucleus (N) and nucleolus (Nu), mitochondria with well-organized cristae and electron-lucent matrix. Rough endoplasmic reticulum (rer) is dispersed within the cytoplasm. The cytoplasm is densely packed with ribosomes and contains some multivesicular bodies (mv) - being a sign of normal cell metabolism.

After treatment with 1 μM compound 3 (Figure 10C) numerous cytoskeleton components (microfilaments) (cs) were present within the cytoplasm. Mitochondria (m) showed a slightly condensed matrix with respect to control cells, mitochondrial cristae remain properly organized. Similar alterations at ultrastructural level were induced by 1 μM C5 complex of vanadium (TEM image not shown). Serious degradation of cell organelles have been observed after treatment of cells with 5 μM compound 3 (Figure 10D), namely numerous degenerative late autophagic vacuoles (avd) have been observed within the perikaryon.

As shown in Figure 10E, PANC-1 cells underwent cell death after treatment with 5 μM of compound 5. The round shape of the cell suggests that it has been detached when the fixative was added. This suggest a process of nonapoptotic cell death, as neither the nucleus (N) nor cytoplasm is condensed. The characteristic shape of heterochromatin, which is organized in threads probably of RNA characteristic for nucleolonema (arrowheads); nucleolus (Nu). Serious degradation of cell organelles have been observed, namely numerous vacuoles, electron-dense lysosomes were present within the pale cytoplasm, being an additional feature of degenerative alterations.

**Figure 11:**
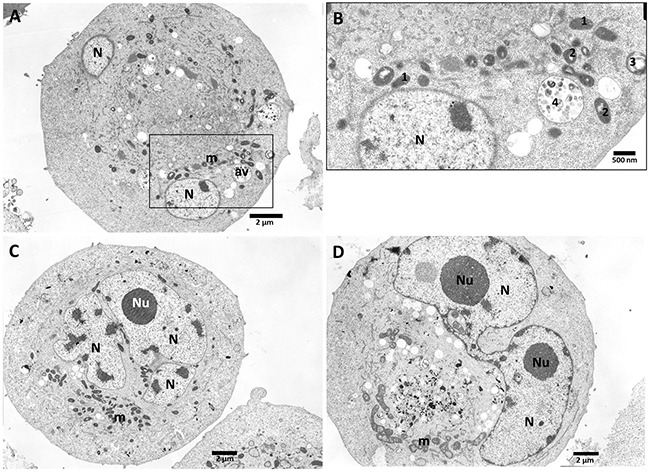
PANC-1 cells treated with vanadium complex C7 (10 μM) Cells showed present double micronuclei (N) which suggests abnormal cell division as a possible result of mitotic catastrophe. **(A)** Atypical, round cell with two cross-sections of nucleus (N), condensed mitochondria (m) and autophagic vacuoles (av). Magnification ×4000. **(B)** (magnified boxed area from A) - Stages of mitochondrial degeneration: 1 – condensed mitochondria, 2 – further degenerative changes within mitochondrial cristae and matrix, (swirling of cristae and dilatations within matrix), 3 – further swirling and folding of mitochondrial membranes, formation of myelinelike bodies, 4 – final formation of autophago-vacuoles (av) after fusion ofmyelinlike structures with some other vacuoles and/or lysosomes. Magnification ×10000. **(C)** Multinucleation and D – the butterfly shape of nucleus (N) suggests that mitosis has failed; condensed mitochondria (m), numerous vacuoles suggest further degenerative processes within the cell. Magnification ×3000.

After treatment with 10 μM of C7 (Figure 11) cells show double nuclei (N) which indicates abnormal cell division as a result of mitotic catastrophe. Atypical, round cell with two cross-sections of nucleus (N), condensed mitochondria (m) and autophagic vacuoles (av) have been observed (Figure 11A). Figure 11B (magnified boxed area from Figure 11A) demonstrates different stages of mitochondrial degeneration. The butterfly shape of nucleus (N) (Figure 11C, 11D) suggests that cell division has not been completed. Condensed mitochondria (m), numerous vacuoles suggest degenerative processes within the cell.

## DISCUSSION

Vanadium compounds, in particular organic deri-vatives, are proposing for the treatment of diabetes, endemic tropical diseases, bacterial infections, HIV infections and cancer [39]. In our study we investigated the effect of seven vanadium compounds on viability of pancreas ductal adenocarcinoma cells (PANC-1) and non-tumor immortalized pancreas duct epithelial cells (hTERT-HPNE). The PANC-1 cell line is one of the most studied and well-characterized *in vitro* models of poorly differentiated human pancreatic adenocarcinoma [40]. Indeed, we found that these complexes significantly decreased pancreatic cancer cell viability. When considering the IC_50_ and selective cytotoxicity against PANC-1 cells, we have chosen three vanadium complexes, containing different organic ligands: derivative of quinolone (C7) and phenanthroline (C3, C5) for further investigation. It has been previously observed that oxovanadium(IV) complexes, derivatives of compound 3 and 5 (Figure 1), exerted a potent *in vitro* cytotoxic activity against different human cancer cells, including brain tumor/glioblastoma, breast, testicular or acute myeloid leukemia cell lines, larynx carcinoma, ovarian carcinoma [41]. Moreover, Wu et al. [42] showed that vanadium compounds exhibit antiproliferative effect against human pancreatic cancer cell line (AsPC-1). Nonetheless, it has not been demonstrated a selective cytotoxicity with use non-tumor cell lines. As far as we know this is the first time when studies present data on selective cytotoxicity of vanadium complexes against pancreatic cancer cell line. It has been documented that oxidative stress and generation of ROS plays a significant role in anticancer activity of vanadium compounds [43]. Indeed, compound 7 at selective concentration (10 μM) caused a marked increase in intracellular ROS level, 7-fold as compared with control cells. This result is consistent with ultrastructural changes in the mitochondria. Some reports have emphasized that mitochondrial alteration may contribute to cell death [44, 45]. It has been noticed that remodeling of mitochondria to the condensed state with dilated cristae junctions is associated with oxidative stress, either caused by elevated ROS production or reduced antioxidant protective mechanisms [46]. Recently, Wu et al. [42] have also observed that ROS generation is implicated in vanadium compounds-induced pancreatic cancer cell death. Similarly, another study showed that the pro-apoptotic activity of *N,N*-ethylenebis (pyridoxylideneiminato) vanadium(IV) complex in human lung carcinoma cells (A549) depends on intracellular ROS increase [47]. On the other hand, Wang et al. [48] demonstrated antitumor activities of vanadium complexes in human hepatoma HepG2 cells without a significant effect on ROS level. Moreover, Scrivens et al. [49] demonstrated that bisperoxovanadium (bpV) compounds exhibited cytotoxic effect in different cancer cells and it seems to be not connected with oxidative stress. Consistent with these results, our data indicated that C3 and C5 at selective concentration (1 μM) (Figure 2) did not cause any changes in ROS level when compared with untreated cells (Figure 5). These compounds at higher concentration (non-selective), which induced cell death in both pancreatic adenocarcinoma cells and in an immortalized pancreatic duct cells, but much stronger in cancer cell, caused a significant elevation in ROS level. Similarly, Cortizo et al. [50] found that vanadium complexes inhibited cellular proliferation through the ROS generation both in osteoblastic cells derived from mouse (MC3T3E1) and osteosarcoma cells (UMR106) from a rat, but the cytotoxicity was stronger in tumor cells.

Previously, it has been suggested that vanadium compounds-induced an increase of ROS level in pancreatic cancer cells may correlate with the G2/M cell cycle arrest [42]. Indeed, we observed that C7 at concentration of 10 μM caused an elevation of ROS level and disrupted cell cycle by a significant increase in the number of cells in the G2/M phase. However, C3 and C5 did affect neither ROS level nor cell cycle at selective concentration of 1 μM. Lack of influence on cell cycle correlated with any changes in cell proliferation (BrdU assay) in PANC-1 cells. On the other hand, vanadium compounds have also been shown to cause the G1/S phase arrest in human liver cancer cell line [51]. Several studies indicated that either generation of ROS or arresting the cell cycle is implicated in vanadium compounds-induced cancer cell death [43, 52, 53]. For example, vanadium treatment caused a prominent chromatin condensation and cell cycle arrest leading to apoptosis in human breast cancer cell line (MCF7) [54]. According to study by Wang et al. [48] vanadium salt (sodium orthovanadate) suppressed the growth of an immortalized hepatic cell line through inducing G2/M cell cycle arrest and apoptosis. Furthermore, Wu et al. [55] found that sodium orthovanadate could inhibit autophagy in hepatocellular carcinoma cells, contributing to its cytotoxic activity and it was associated with G2/M cell cycle arrest.

In our study we used necrostatin-1 and Z-VAD-FMK (caspase inhibitor) to determine type of programmed cell death in PANC-1 induced by vanadium complexes. Necrostatin-1 because of it specific inhibitory effect of RIP1, was defined as an inhibitor of necroptosis [56]. The inactivation of RIP1 blocks interaction between RIP3 and RIP1, thus prevents the formation of necrosome, which is needed to trigger necroptosis process [57]. Necrostatin-1 is commonly used to determine necroptosis, also in PANC-1 cell line [58]. Besides, RIP-1 kinase is involved in activation of nuclear factor (NF)-κB signaling as well as trigger of apoptosis. When caspase 8 is activated, RIP1 and RIP3 are cleaved under apoptotic conditions, thereby preventing RIP1-miediated necroptosis activation [59]. In case of blocking apoptotic cell death by pan-caspase inhibitors, necroptosis is used as an alternative cell pathway [60]. We have found that either necrostatin-1 or Z-VAD-FMK protected against C3- and C5-induced PANC-1 cell death measured by MTT assay (Figure 7). This result suggested that C3 and C5 complexes induced a mix type of cell death, including apoptotic and necroptotic process. Indeed, we have observed a significant increase of apoptotic/necroptotic (Annexin V+/PI+) cells population after treatment of PANC-1 cells with non-selective concentration of C3 and C5. Lack of significant elevation of apoptotic/necroptotic cells after exposure to selective concentration of C3 and C5 was probably caused by differences in sensitivity and specificity of using techniques [61]. MTT assay evaluates decrease of cells viability associated with alteration in mitochondrial metabolism [62]. Furthermore, C3 and C5 at non-selective concentration of 5 μM slightly increased the sub-G1 population in PANC-1 and this results indicated only a limited extent of apoptosis in cell death. Besides, we determined an increase of pro-apoptotic Bax protein level after exposure to C3 or C5 at selective and non-selective concentrations, as well as an decrease of anti-apoptotic Bcl-2 protein level for all investigated complexes at selective and non-selective concentrations. Interestingly, we have also found that RIP1 and RIP3, which are markers of necroptosis, increased after treatment with C3 and C5 complexes in a concentration-dependent manner. Thus, there arise the question: How can we explain the presence of pro-apoptotic and necroptotic markers in PANC-1 cells together after exposure to vanadium complexes? It has been documented that increased of Bax level can promoting apoptotic and also necroptotic process [63]. Moreover, it has been found that Bcl-2, antiapoptotic protein, can suppress not only apoptosis but also necroptosis as well as autophagy [64]. Above findings are corresponding to our results. Additionally, we have observed ultrastructural changes in PANC-1 cells induced by C3 and C5 complexes in concentration-depended manner. Even the subtle changes, induced by 1 μM of C3 and C5, may cause important alteration in cell function [65]. Our TEM analysis suggested a process of nonapoptotic cell death, as neither the nucleus nor cytoplasm is condensed. Neither the blebbing phenomenon have been observed. Moreover, we found that C5-treated PANC-1 cells showed incomplete chromatin condensation, in opposite to apoptosis process, while caspase-dependent strong chromatin compaction is observed [66]. Interestingly, we have found autophagic vacuoles, which indicated autophagy process after treatment of PANC-1 cells with higher, non-selective concentration of C3 and C5 as well as C7 at all investigated concentrations. However, C3 and C5 at 1 μM decreased the level of LC3, indicating that inhibition of autophagy process could be responsible for a significant decrease in PANC-1 cell viability. It has been proven that autophagy in PANC-1 cells plays a protection function against cell death and it is connected with chemoresistance of pancreatic cancer [67]. An inhibition of autophagy process in pancreatic cancer cells augmented cytotoxicity of commonly used chemotherapeutics, such as gemcitabine and 5-fluorouracil [68]. From a therapeutic perspective it is really beneficial mechanism of action and hydroxychloroquine, autophagy inhibitor, has been approved for clinical investigation [69]. On the other hand, the higher level of LC3-II protein induced by 5 μM C3 and C5 complexes could be connected with necroptosis process. Autophagic vesicles are commonly observed in necroptotic cells and it is suggested that autophagy could be execution mechanism in necroptosis process [70].

Interestingly, C7 complex caused the cell cycle arrest in G2/M phase associated with multinucleation and binucleation, suppression of cell proliferation and an increase of ROS level simultaneously with autophagy process. Micronucleation, double nuclei as well as multinucleated cells is a morphological feature of mitotic catastrophe [71–73]. This results correlated with a significant decrease of PANC-1 cell viability after 48 h of treatment. Indeed, it has been reported the presence of autophagic vacuoles during mitotic catastrophe process [74]. However, the role of autophagy in mitotic catastrophe is still not clarified. [75]. In general, mitotic catastrophe is not considered a form of cell death, nonetheless it is indicated as an irreversible trigger for cells death [76].

To summarize our research results, we indicated that vanadium complexes exhibited a selective cytotoxic activity against the pancreas ductal adenocarcinoma cells, throughout different type of cell death, depend on chemical structure and concentration. Most important, we have shown, for the first time, that vanadium complexes induced selective toxicity in pancreatic cancer cells in comparison to non-tumor cells of the same tissue. What is more, we found that C3 and C5 inhibited autophagy process in selective concentration, whereas at higher - trigger necroptotic pathway. It has never been demonstrated before that vanadium compounds could induce necroptosis in cancer cells. It is going to be really important that potential anticancer compounds be able to trigger alternative type of cell deaths to apoptosis-resistant cancer cells. It is also worth stressing that C7 caused PANC-1 cells death through mitotic catastrophe in presence of autophagic vacuoles, despite the similar structure with C5, containing only different organic ligands. Different cytotoxic effect on PANC-1 cells emphasize that the type of cell death induce by vanadium complexes is mainly determined by ligands. Moreover, organic component affect the selective activity of vanadium complexes if we compare structure C2-C7 with C1- pure vanadium salt.

Taking the above conclusion about structure-activity relationship and type of cell death induced by vanadium complexes it is highly recommended further studies supporting the therapeutic potential of vanadium in pancreatic cancer treatment.

## MATERIALS AND METHODS

### Reagents

In this study was used: anti-rabbit and anti-goat secondary antibodies as well as polyclonal RIP1, RIP3, Bax and Bcl-2 antibody from Santa Cruz Biotechnology (Santa Cruz, CA); polyclonal LC3 antibody from Abcam; 3-(4,5-dimethylthiazol-2-yl)-2,5-diphenyltetrazolium bromide (MTT) and RNAse A and 2′,7′-Dichlorofluorescin diacetate (DCF-DA) from Sigma-Aldrich; Annexin V-FITC apoptosis detection kit and propidium iodide staining solution from BD Pharmingen; BrdU incorporation ELISA assay from Roche (Grenzach-Wyhlen, Germany).

### Chemistry

The reagents (Sigma-Aldrich) used for the syntheses were of analytical grade and were used without further purification. They were as follows: VO(acac)2 (≥98%), VOSO_4_ (≥99.99% trace metals basis), 2,2′-oxydiacetic acid (H_2_oda) (≥98%), 2,2′-thiodiacetic acid (H_2_tda) (≥98%), nitrilotriacetic acid (H_3_nta) (≥99%), 1,10-phenanthroline monohydrate (phen, reagent grade), 2,2′-bipyridine (bpy, ≥98%) and 4-amino-2-methylquinoline (4-NH_2_-2-Me(Q), ≥98%).

The synthesis of [VO(oda)(H_2_O)_2_] (Compound 2) was carried out using a procedure reported in literature [77]. The syntheses of [VO(oda)(phen)](H_2_O)_1.5_ (Compound 3) and [VO(tda)(bpy)](H_2_O)_1.5_ (Compound 4) were carried out according to the procedures described in the literature [78].

The synthesis of [phenH][VO(nta)(H_2_O)](H_2_O)_0.5_ (Compound 5), [bpyH][VO(nta)(H_2_O)](H_2_O)_1.5_ (Compound 6) and [4-NH_2_-2-Me(QH)][VO(nta)(H_2_O)](H_2_O) (Compound 7) was performed according with follow procedure: the mixture of VO(acac)_2_ (10 mmol) and H_3_nta (10 mmol) in water (40 mL) was refluxed for ca. 0.5 h. The hot solution was filtered and cooled. To this solution, an methanolic solution of 1,10-phenanthroline monohydrate, 2,2′-bipyridyl (10 mmol) or 4-amino-2-methylquinoline was added. Then, the mixture was concentrated (in order to eliminate Hacac- acetylacetone by evaporation) and left for a crystallization at the room temperature. After 5-7 days a blue precipitate of the complex fell out. The recrystallization from hot water gave blue crystals after 4-10 days. The crystals were air-dried at the room temperature. Experimental details of the synthetic methodology, IR spectra, elemental analysis as well as reaction schemes have been included in the Supplementary Materials (Supplementary Figure 1).

The compositions, structure and purity of the all synthesized vanadium complexes, including new synthesized [phenH][VO(nta)(H_2_O)](H_2_O)_0.5_ (Compound 5), [bpyH][VO(nta)(H_2_O)](H2O)_1.5_ (compound 6) and [4-NH_2_-2-Me(QH)][VO(nta)(H_2_O)](H_2_O) (compound 7) were established on the basis of the elemental analysis of carbon, hydrogen, nitrogen and sulphur (Vario EL analyzer Cube CHNS) as well as the IR spectra, which were recorded on a BRUKER IFS 66 spectrophotometer in a KBr pellet over the 4400–650 cm^−1^ range.

For each experiments were prepared fresh aqueous solutions of vanadium compounds at initial concentration of 0.5 mM (stock solution). All solution was filtered through a 0.22 μm filter and diluted in serum-free culture medium to the appropriate concentration.

### Cell culture

The pancreas ductal adenocarcinoma cell lines (PANC-1) and immortalized pancreas duct cells (hTERT-HPNE) were purchased from the American Type Culture Collections (ATCC). PANC-1 cells were cultured in Dulbecco's Modified Eagle Medium (DMEM) with high glucose concentration (4.5 mg/ml), supplemented with 100 units/ml penicillin, 100 μg/ml streptomycin and 10% fetal bovine serum (FBS), while hTERT-HPNE cells were cultivated in medium composed of three volumes of glucose-free DMEM, one volume of medium M3, 5.5 mM glucose, 5 ng/ml EGF, 2 mM glutamate, 750 ng/ml puromycin and 5% FBS. Cell cultures were incubated in a humidified atmosphere of 95% air and 5% CO_2_ at 37°C. All experiments were performed on cells with 80-90% confluence.

### Cell viability assay

Cell viability was measured by MTT assay according to the protocol. Briefly, PANC-1 and hTERT-HPNE cells ware seeded on 96-well plates at a density of 14-16×10^3^ cells/100 μl. On the next day, cells were treated with vanadium complexes (1-100 μM) dissolved in serum-free medium and incubated for 48h. Then, tetrazolium dye (MTT) was added to medium and after 2 hours the absorbance was measured at 492 nm. The data were expressed as the percentage of untreated cells (control), which was set to 100%.

In another investigation, Z-VAD-fmk (caspase inhibitor) and Necrostatin-1 were dissolved in DMSO at concentration of 40 μM and 10 μM, respectively, and added 1h prior the treatment with vanadium complexes (1-100 μM). After 48 h, cell viability was determined on the base of untreated cells in the presence of DMSO (0.1%) as a control. A blank absorbance values, as assessed from cell-free wells, were subtracted from the absorption values of each test sample.

### Cell proliferation

PANC-1 cells were seeded at concentration of 14-16×10^3^ cells per well in 96-well plate in concentration range: 1-10 μM for C3,C5 and 1-25 μM for C7. The following day, cells were exposed to vanadium complexes in Free serum (FS) conditions. After 48h incubation, the antiproliferative activity was measured by a BrdU incorporation, ELISA assay, according to the protocol. Briefly, cells were incubated with BrdU solution for 2 hours, fixed for 30 min by FixDenat solution and then incubation with an anti-BrdU-POD conjugate antibody for 90 min at room temperature. In next step, the cells were rinsed three times and substrate solution was added. After 20 min the reaction was terminated with 1M H_2_SO_4_ and the absorbance was measured at 450 nm. The data were expressed as the percentage of untreated cells (control), which was set to 100%.

### Detection of ROS

The generation of intracellular ROS level was determined by flow cytometry technique. PANC-1 cells were seeded into 6-well plates at a density of 14-16×10^3^ and the next day, cells were treated by vanadium complexes in concentration range: 1-5 μM for C3, C5 and 1-10 μM for C7. After of 48h incubation the cells were exposed to 10 μM 2,7-dichlorofluorescein diacetate (DCF-DA) for 30 min at 37°C. Fluorescence of oxidized DCF was measured by flow cytometer at an excitation wavelength of 480 nm and an emission wavelength of 525 nm. The data were expressed as the percentage of untreated cells (control), which was set to 100%.

### Analysis of apoptosis

The apoptosis detection was performed by using the Annexin V-FITC apoptosis detection kit and according to manufacturer's instructions. Briefly, PANC-1 cells were seeded into 6-well plates (10^6^ cells per well). After 48h treatment with vanadium complexes at concentration of 1, 5 μM for C3, C5 and 10, 15 μM for C7, cells were collected, washed twice with ice-cold PBS, pelleted and resuspended in binding buffer (50 mM HEPES). Subsequently, 5 μl of Annexin V and 2.5 μl of propidium iodide (PI) were added to the cells and it was incubated in the dark for 15 min at room temperature. After incubation, cells were analyzed by flow cytometry within 1 h. Ten thousand specific events were analyzed. Data were expressed as a percentage of total population.

### Cell cycle analysis

PANC-1 cells were seeded into 6-well plates and exposed to vanadium complexes (C3, C5, C7) for 48 h in selected concentrations: 1, 5 μM for C3,C5 and 10, 15μM for C7. After this, cells were harvested, washed twice with ice-cold PBS and fixed with 70% ethanol at 4°C overnight. The next day, the ethanol was removed by centrifugation and cells were resuspended in PBS containing RNAse A (50 μg/ml) and PI (50 μg/ml). After 30 min incubation in the dark, cells were analyzed by flow cytometer. The analysis has been performed using CellQuest Pro software. At least 10 000 cells were collected. Debris and doublets have been removed by gated appropriate population on FSC/SSC and FL2-A/FL2-W plots before analysis. The percentage of cells in each cell cycle phase was determined by using markers set within the analysis program.

### Western blot analysis

Western blot analysis was performed in order to investigate Bax, RIP1, RIP3, LC3, Bcl-2 according to previous described protocol [79]. PANC-1 cells were seeded into 100mm petri dishes and cultured until reached about 90% confluence. After this, cells were incubated with vanadium complexes (C3,C5 and C7) at concentration of 1, 5, 10 and 15 μM for 48 h. Subsequently, conditioned media were discharged and attached cells washed with PBS, detached, and after all homogenized. After electrophoresis, proteins were transferred onto nitrocellulose membrane (Protran®, Schleicher and Schuell BioScience) and detected using antibodies: anti-Bax, anti-RIP1, anti-RIP3, anti-LC3 and anti-Bcl-2. β-actin was used as a loading control. Protein levels were quantified using densitometry software (ImageQuant Software)

### Transmission electron microscope

Cells growing on 100 mm petri dishes were incubated with vanadium complexes 3,5 and 7 by 48 h in different concentration (1-10 μM). After this, cells were fixed in 2.5% glutaraldehyde in 0.1 mM sodium-cacodylate buffer. Next fixed cells were detached by scraping and centrifuged. The cell pellets were postfixated in 2% osmium tetroxide and briefly dehydratated in graded series of ethanol. After infiltration with propylene dioxide: epon mixture and pure epon pelleted cells were embedded to polymerize. Finally the ultra-thin sections (Reichert OmU3 ultramicrotome, Austria) were contrasted using uranyl acetate and lead citrate prior to examination in transmission electron microscope at 100kV (JEM 1200EX II, Jeol, Japan).

### Statistical analysis

The obtained data are reported as the mean ± SD for triplicate determination of 3 separate experiments. The log IC_50_ was calculated using the GraphPad Prism 5 program (GraphPad) by non-linear regression analysis: log(inhibitor) vs. normalized response (Supplementary Figure 2). Each compound of vanadium or gemcitabine was tested at least in triplicate.

## SUPPLEMENTARY FIGURES


