# Supplementary material for: Selective cytotoxicity of vanadium complexes on human pancreatic ductal adenocarcinoma cell line by inducing necroptosis, apoptosis and mitotic catastrophe process
Source: Oncotarget. 2017 Jul 22;8(36):60324–41. doi: 10.18632/oncotarget.19454 (PMC5601142; doi:10.18632/oncotarget.19454)
Supplement: Supplementary file 1 [file oncotarget-08-60324-s001.pdf]

Selective cytotoxicity of vanadium complexes on human pancreatic ductal adenocarcinoma cell line by inducing necroptosis, apoptosis and mitotic catastrophe process

SUPPLEMENTARY MATERIALS

Compound 2 (C2): [VO(oda)(H_2O)$_2$]

The synthesis [VO(oda)(H_2O)$_2$] was carried out using a procedure reported in literature [1]. The mixture of stoichiometric quantities of VO(acac)$_2$ (acac = acetylacetonate) and H_2oda (15 mmol each) in 50 mL of water was refluxed for ca 3 hours. Then, the hot mixture was filtered, concentrated to a volume of about 25 mL (in order to eliminate Hacac by evaporation) and slowly cooled. Blue crystals of [VO(oda)(H_2O)$_2$] were filtered off, washed with acetone and diethyl ether and dried in a vacuum desiccator over P_4O_{10}. Anal. Calcd for [VO(oda)(H_2O)$_2$] C, 20.44%, H, 3.44%: Found: C, 20.42%, H, 3.45%. A strong bands at ca. 1588 and 1430 cm^{-1} can be assigned to antisymmetric and symmetric vibration of the COO$^-$ groups, respectively. The IR spectrum of the complex displays the band at ca. 1140 cm^{-1} corresponding to the antisymmetric COC stretching that is characteristic for the *mer* conformation of the oda ligand.

Compound 3 (C3): [VO(oda)(phen)](H_2O)$_{1.5}$

Compound 4 (C4): [VO(tda)(bpy)](H_2O)$_{1.5}$

The syntheses of [VO(oda)(phen)](H_2O)$_{1.5}$ and [VO(tda)(bpy)](H_2O)$_{1.5}$ were carried out according to the procedures described in the literature [2]. 4H_2O and [V(O)(oda)(phen)]. 1.5H_2O.*Polyhedron* 29 (2010) 3028–3035]. Anal. Calcd for [VO(oda)(phen)](H_2O)$_{1.5}$: C, 47.27 %, H, 3.69 %, N, 6.9%, Found: C, 47.17 %, H, 3.64 %, N, 7.1%; [VO(tda)(bpy)](H_2O)$_{1.5}$: C, 42.21%, H, 3.01%, N, 7.03%, S, 8.04%, Found: C, 41.29%, H, 3.96%, N, 6.86%, S, 7.87%. IR spectra display a very strong and broad absorption bands at about 1595 cm^{-1} and 1615 cm^{-1}, respectively for [VO(oda)(phen)]1.5H_2O and [VO(tda)(bpy)]1.5H_2O, which correspond to the antisymmetric vibrations of the oda and tda carboxylate groups. Characteristic for oxidovanadium(IV) complexes bands at 974 cm^{-1} and 978 cm^{-1}, respectively for VO(oda)(phen)]. 1.5H_2O and [VO(tda)(bpy)]. 1.5H_2O can be assigned to the V=O stretching mode [3]. The frequencies for [VO(oda)(phen)]. 1.5H_2O: 1521 cm^{-1} phen - ν_{ring}, 1428 cm^{-1} phen - ν_{ring} + $\delta_{ring\text{-}H}$, 1226 cm^{-1} and 1110 cm^{-1}, phen - $\delta(CH)_{in\ plane}$. The frequencies for [VO(tda)(bpy)]. 1.5H_2O: 1496 cm^{-1} and 1471 cm^{-1} bipy - ν_{ring}, 1444 cm^{-1} bipy - ν_{ring} + $\delta_{ring\text{-}H}$, 1281 cm^{-1}, 1235 cm^{-1}, 1059 cm^{-1} and 1035 cm^{-1} bipy - $\delta(CH)_{in\ plane}$. In the IR spectra of both complexes, OH stretching bands of lattice water are presented at ca 3500 – 3110 cm^{-1}.

Compound 5 (C5): [phenH][VO(nta)(H_2O)](H_2O)$_{0.5}$

Compound 6 (C6): [bpyH][VO(nta)(H_2O)](H_2O)$_{1.5}$

Compound 7 (C7): [4-NH_2-2-Me(QH)][VO(nta)(H_2O)](H_2O)

The syntheses were carried out according to the procedures described in the literature [4, 5]. The mixture of VO(acac)$_2$ (10 mmol) and H_3nta (10 mmol) in water (40 mL) was refluxed for ca. 0.5 h. The hot solution was filtered and cooled. To this solution, an methanolic solution of 1,10-phenanthroline monohydrate, 2,2′-bipyridyl (10 mmol) or 4-amino-2-methylquinoline was added. Then, the mixture was concentrated (in order to eliminate Hacac by evaporation) and left for a crystallization at the room temperature. After 5-7 days a blue precipitate of the complex fell out. The recrystallization from hot water gave blue crystals after 4-10 days. The crystals were air-dried at the room temperature. Anal. Calcd for [phenH][VO(nta)(H_2O)](H_2O)$_{0.5}$: C, 46.6%, H, 3.7%, N, 9.1%, Found: C, 46.1%, H, 3.9%, N, 9.0%; [bpyH][VO(nta)(H_2O)](H_2O)$_{1.5}$: C, 42.9%, H, 4.3%, N, 9.4%, Found: C, 42.7%, H, 4.3%, N, 9.3%.; [4-NH_2-2-Me(QH)][VO(nta)(H_2O)](H_2O): C, 42.66%, H, 4.71%, N, 9.33%, Found: C, 42.44%, H, 4.83%, N, 9.27%. Aqueous solutions of these compounds have shown a high stability, e.g. being resistant to the oxidation in air, i.e. remain unaltered (UV–Vis control) for at least 3 days. The characteristic for the oxidovanadium(IV) compounds band at 981, 978 and 980 cm^{-1} can be assigned to the V=O stretching mode for [phenH][VO(nta)(H_2O)](H_2O)$_{0.5}$, [bpyH][VO(nta)(H_2O)](H_2O)$_{1.5}$ and [4-NH_2-2-Me(QH)][VO(nta)(H_2O)](H_2O), respectively. Two bands at 1586-1595 and 1402-1400 cm^{-1} correspond to the antisymmetric and symmetric vibrations of the ionized COO$^-$ groups, respectively. This finding confirms the contribution of the carboxylate groups in the coordination of V(IV) in a monomeric [VO(nta)(H_2O)]$^-$ coordination entity. The difference, $\Delta\nu$, between the frequencies of antisymmetric [ν_{as}(OCO$^-$)] and symmetrical [ν_s(OCO$^-$)] vibrations for carboxylate group in the compounds and in the nitrilotriacetate sodium salt, Na_3nta, ($\Delta\nu$ = 1598 – 1406 = 192 cm^{-1}) suggests the ionic character of the VO-nta interactions [6]. The bands at 1090 - 1095 cm^{-1} that can be assigned to the stretching vibrationν(C-N) of the nta ligands [7] is shifted ca. 100 cm^{-1}

in relation to *ν*(C-N) in the free H_3nta (1200 cm^{-1}). It indicates that the N atom of the nta ligand coordinates to V atom. The presence of the stretching vibration bands at 3464 – 3485 cm^{-1} indicates the attachment of a proton to the nitrogen atom of bpy, phen and 4-NH_2-2-Me(Q). Moreover, the IR spectra of the compounds show bands at 3300 – 3100 cm^{-1} and 1660 – 1610 cm^{-1} that can be assigned to antisymmetric and symmetric OH stretching and HOH bending bands of the lattice and coordination water, respectively.

REFERENCES

1. del Río D, Galindo A, Tejedo J, Bedoya FJ, Ienco A, Mealli C. Synthesis, antiapoptotic biological activity and structure of an oxo–vanadium (IV) complex with an OOO ligand donor set. Inorg Chem Commun. 2000; 3: 32-34.
2. Álvarez L, Grirrane A, Moyano R, Álvarez E, Pastor A, Galindo A. Comparison of the coordination capabilities of thiodiacetate and oxydiacetate ligands through the X-ray characterization and DFT studies of [V (O)(tda)(phen)]· 4H 2 O and [V (O)(oda)(phen)]· 1.5 H 2 O. Polyhedron. 2010; 29: 3028-3035.
3. Banik B, Somyajit K, Nagaraju G, Chakravarty AR. Oxovanadium(IV) complexes of curcumin for cellular imaging and mitochondria targeted photocytotoxicity. Dalton Trans. 2014; 43: 13358-13369.
4. Tesmar A, Inkielewicz-Stępniak I, Sikorski A, Wyrzykowski D, Jacewicz D, Zięba P, Pranczk J, Ossowski T, Chmurzyński L. Structure, physicochemical and biological properties of new complex salt of aqua-(nitrilotriacetato-N,O,O’,O’’)-oxidovanadium(IV) anion with 1,10-phenanthrolinium cation. J. Inorg. Biochem. 2015; 152: 53-61.
5. Tesmar A, Wyrzykowski D, Kruszyński R, Niska K, Inkielewicz-Stępniak I, Drzeżdżon J, Jacewicz D, Chmurzyński L. Characterization and cytotoxic effect of aqua-(2,2′,2″-nitrilotriacetato)-oxo-vanadium salts on human osteosarcoma cells. Biometals. 2017; 30: 261-275.
6. Nakamoto K. Infrared and Raman Spectra of Inorganic and Coordination Compounds Part B: Applications in Coordination, Organometallic, and Bioinorganic Chemistry. John Wiley & Sons, Inc. 2009; 289-290.
7. Tomita Y, Ueno K. The properties and infrared absorption spectra of nitrilotriacetate chelates. Bull Chem Soc Jpn. 1963; 36: 1069-1073.

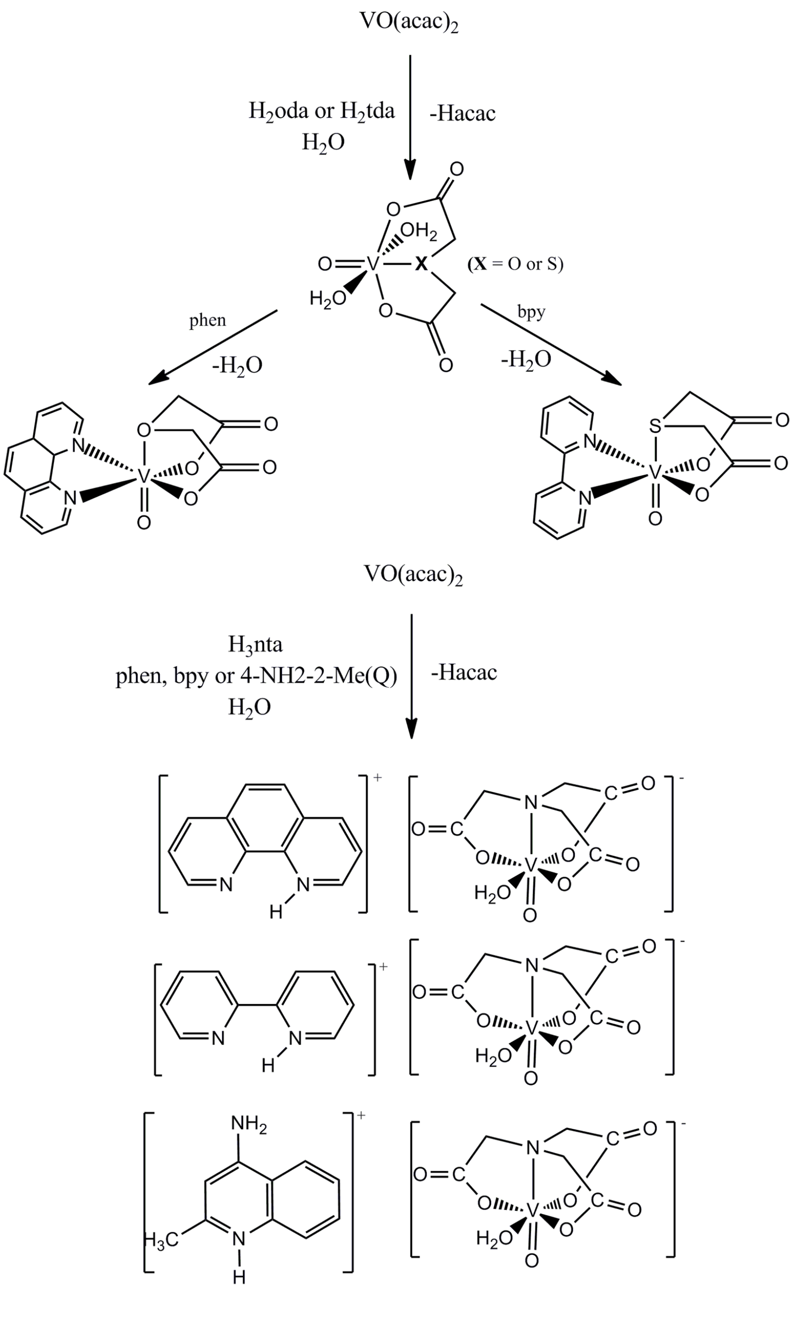

Supplementary Figure 1: Scheme of synthesis of vanadium complexes.

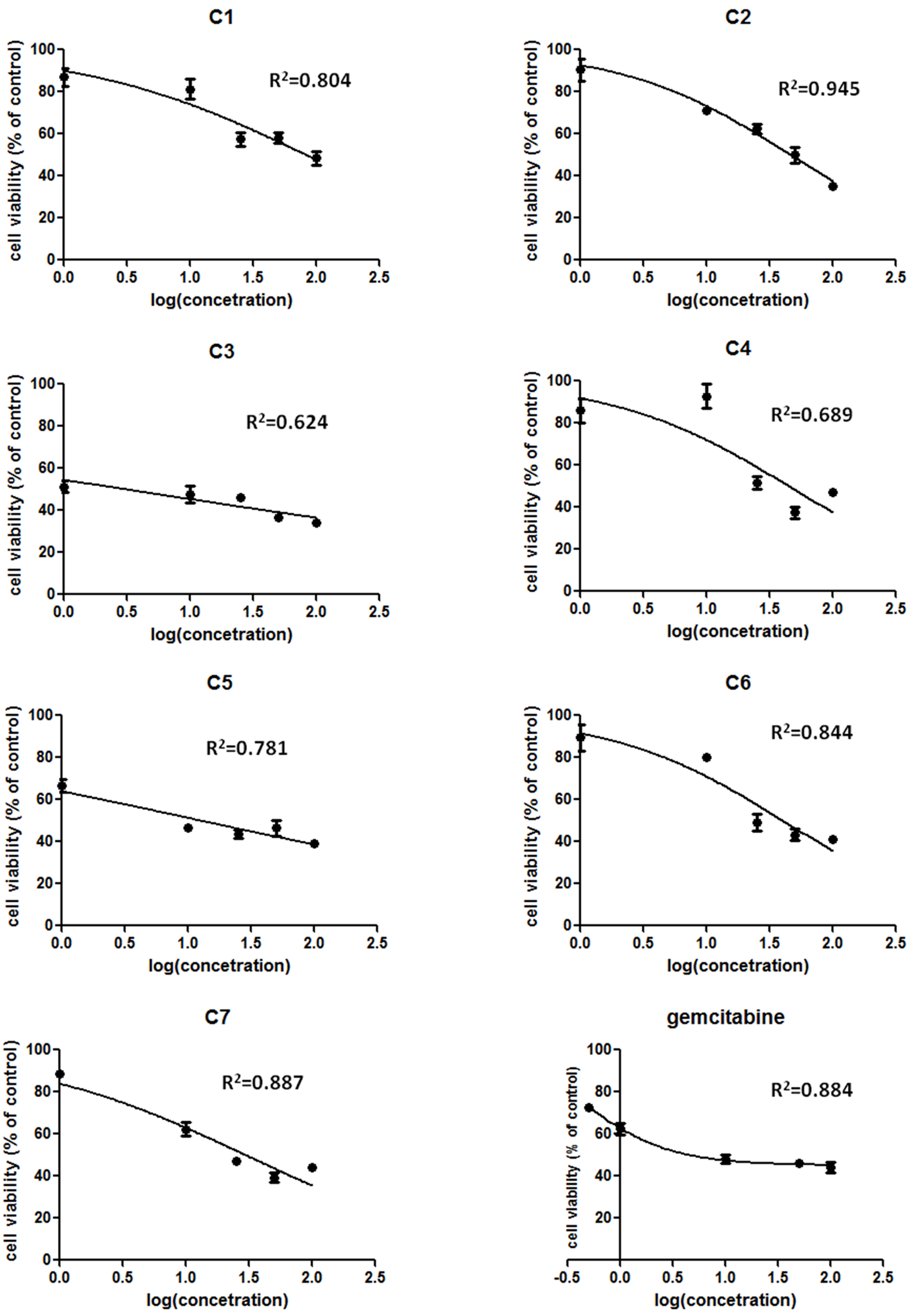

Supplementary Figure 2: Log(concentration) vs. cell viability plots. Non-linear regression analysis: log(inhibitor) vs. normalized response has been performed to calculate log IC_{50} values. The obtained data are reported as the mean ± SD for triplicate determination of 3 separate experiments. R^2- coefficient of determination.
